# ‘If there were doctors who could understand our problems, I would already be better’: dissatisfactory health care and marginalisation in superdiverse neighbourhoods.

**DOI:** 10.1111/1467-9566.13061

**Published:** 2020-02-04

**Authors:** Hannah Bradby, Antje Lindenmeyer, Jenny Phillimore, Beatriz Padilla, Tilman Brand

**Affiliations:** ^1^ Sociology Department Uppsala University Uppsala Sweden; ^2^ Institute of Clinical Sciences University of Birmingham Birmingham UK; ^3^ Institute for Research into Superdiversity (IRiS) School of Social Policy University of Birmingham Birmingham UK; ^4^ Department of Sociology University of South Florida Tampa USA; ^5^ Instituto Universitario de Lisboa (ISCTE‐IUL) Lisbon Portugal; ^6^ Department Prevention and Evaluation Leibniz Institute for Prevention Research and Epidemiology – BIPS Bremen Germany

**Keywords:** diversity, healthcare, user perspectives, service improvement, access, quality of care, disappointment, dissatisfaction

## Abstract

How people in community settings describe their experience of disappointing health care, and their responses to such dissatisfaction, sheds light on the role of marginalisation and underlines the need for radically responsive service provision. Making the case for studying unprompted accounts of dissatisfaction with healthcare provision, this is an original analysis of 71 semi‐structured interviews with healthcare users in superdiverse neighbourhoods in four European cities. Healthcare users spontaneously express disappointment with services that dismiss their concerns and fail to attend to their priorities. Analysing characteristics of these healthcare users show that no single aspect of marginalisation shapes the expression of disappointment. In response to disappointing health care, users sought out alternative services and to persuade reluctant service providers, and they withdrew from services, in order to access more suitable health care and to achieve personal vindication. Promoting normative quality standards for diverse and diversifying populations that access care from a range of public and private service providers is in tension with prioritising services that are responsive to individual priorities. Without an effort towards radically responsive service provision, the ideal of universal access on the basis of need gives way to normative service provision.

## Introduction

This article offers qualitative evidence from diverse communities in four European settings on how users of health care express dissatisfaction with services and how they respond to disappointing encounters. Healthcare users’ dissatisfaction with and response to negative healthcare experience are a key source of evidence to enable the improvement of the quality of service. Research has prioritised professional perspectives on the provision of services to local clinical populations, and restricted samples of service users to those who can speak the national language, understand the local health system, possess the financial means where necessary and are not mentally distressed. This article offers an analysis of semi‐structured interview data with service users residing in neighbourhoods characterised by migration‐driven diversity, collected as part of a broader study of welfare bricolage (Phillimore *et al*. 2018, 2019a). The article offers a new perspective on the role of healthcare users’ accounts of disappointment and dissatisfaction in improving service provision. We argue that aspects of marginalisation impeding service users from obtaining appropriate healthcare intersect in complex ways. Aspects of marginalisation include poverty, refugee or migration status and language barriers, which recur in various combinations such that no single factor is strongly associated with the expression of disappointment. This implies the need for service provision that is radically responsive to users’ own perspectives and priorities.

The article covers: (1) the wider study as context for this analysis; (2) a review of patient dissatisfaction research to date including the serious existential threat that disappointing service can represent to wellbeing and the limitations of that research; (3) description of the research and analytic methods and (4) reasons people gave for dissatisfaction, and strategies they deployed in response to disappointing health care.

### Study context

This article draws on interview data from the UPWEB study (Phillimore *et al*. 2015) of how residents of diverse neighbourhoods seek health care to address their self‐defined health concerns, conceptualised as welfare bricolage (Phillimore *et al*. 2018). The heuristic of bricolage is understood as tactics of utilising available resources to address health issues. Making bricolage tactics visible can disrupt professional‐centred views of service provision. Our analyses have explored the enormous complexity of the intersecting efforts of individuals and organisations, working in both formal and informal provision in superdiverse neighbourhoods (Pemberton *et al*. 2019). Professionals and civil society organisations re‐purpose existing resources to address gaps in public health provision (Phillimore *et al*. 2019a) with healthcare users also mediating with and supplementing available services for themselves and others (Phillimore *et al*. 2019b). Such work is often unseen and unacknowledged and highly gendered (Bradby *et al*. 2019a). Some women, marginalised by migration, austerity and xenophobia with their entitlement to services questioned, have great difficulty in expressing criticism of the public health system, even when in receipt of highly inadequate services (Bradby *et al*. 2018). This article considers negative healthcare experience, attendant dissatisfaction, how such disappointment is understood and responded to and related to aspects of marginalisation.

### Patient dissatisfaction research

Attention to patient^1^ dissatisfaction is a well‐established route to improving the quality of health care (Doyle *et al*. 2013). Additional difficulties that patients from marginalised and minority backgrounds might experience in accessing appropriate health care (Johnstone and Kanitsaki 2006) and how their negative experiences should be understood (Suurmond *et al*. 2011), remain under‐researched.

The measurement of patient satisfaction involves conceptual and technical challenges (Coulter *et al*. 2014, Funk *et al*. 2012, Jenkinson *et al*. 2002), not least the contradiction of high levels of satisfaction, while reporting failed services (Rogers *et al*. 2000). Assessing patient dissatisfaction tends to give ‘a better picture of the reality’ on which to build efforts ‘to improve the quality of care’ (Eriksson and Svedlund 2007: 439).

Patients who are marginalised in terms of migration, education and employment status, gender, local language ability and mental health and whose entitlement to health care is challenged, can find it difficult even to express dissatisfaction (Bradby *et al*. 2018). Capturing disappointment and dissatisfaction with healthcare experience among highly diverse samples of users, particularly when some people's entitlement is politically contested, represents a significant challenge. The distribution of healthcare resources according to need, regardless of individual (e.g. language ability) and institutional (e.g. healthcare professionals’ time restrictions) barriers (Cattacin *et al*. 2013), could be supported by understanding how patients interpret their disappointment/dissatisfaction and what motivates their responses.

Dissatisfaction is understood as a

‘subjective transformation, which … involves the crystallisation of a strong, undifferentiated, vague, negative emotion experienced immediately (after an untoward event/experience) into a more stable negative interpretation of the experience’ (Coyle and Williams 1999: 8).

We use the terms disappointment and dissatisfaction as two highly related aspects consequent on negative healthcare experience. Negative healthcare experiences do not necessarily or usually translate into the explicit expression of dissatisfaction, given the psycho‐social pressures for patients to re‐evaluate negative experiences of care more positively with the passage of time (Edwards *et al*. 2004) for various reasons (Funk *et al*. 2012), including power relations and victim blaming. Formal complaints are an atypical response to unsatisfactory health care (Allsop and Jones 2008), and are even less common among marginalised migrant patients than among the general population (Garrett *et al*. 2008). Negative encounters that are recalled spontaneously play an important role as ‘critical episodes’ that have shaped healthcare users’ expectations of and attitudes to healthcare provision (Feldmann *et al*. 2007). Our interest is not in interrogating the reliability of healthcare users’ retrospective accounts of negative healthcare episodes (Werner and Malterud 2003), but rather in exploring what they mean for improving services.

Dissatisfied patients may seek alternative advice and/or treatment, and/or withdraw from future healthcare encounters (Annandale and Hunt 1998, Coyle 1999, Mulcahy and Tritter 1998). Women seem more likely than men to express dissatisfaction as part of the gendered family role (Coyle 1999) that includes mediating with healthcare providers (Bertakis *et al*. 2000). Low levels of trust and delimited social participation may increase patients’ levels of dissatisfaction (Lindström and Axén 2004), and patients seen as lacking entitlement (Hill 2010), who have lost hope and trust (Bradby *et al*. 2018) may be more disposed to withdraw from services.

Disappointing health care threatens personal identity, being associated with feeling dehumanised, disempowered and devalued (Coyle 1999); threats that may be more acute for marginalised groups (Hill 2010). This article examines how healthcare users from four different European countries respond to the experience of disappointing care, to trace connections between the interpretations of such experiences and subsequent responses, to understand the influence of aspects of marginalisation.

## Limitations of the research to date

Research on dis/satisfaction has tended to be restricted to single national healthcare systems (Bankauskaite 2003, Carmel 1988, Renzi *et al*. 2002), sampling clinical populations (Doyle *et al*. 2013); often hospital inpatients (Carmel 1988, Rozenblum *et al*. 2013) or primary care settings (Barry *et al*. 2001, Coulter 2005). Specific public services tend to be examined, rather than the mixture of public and private that reflects the day‐to‐day functioning of healthcare systems across Europe (Phillimore *et al*. 2019b), with physicians’ (Wofford *et al*. 2004) or nurses’ (Palese *et al*. 2017) priorities guiding the research (Hill 2010). Professional agendas (Renzi *et al*. 2002) guide the testing of patient responses to specialist (Mangset *et al*. 2008) intervention (Scott *et al*. 2010), rather than assessing all the health care a person experiences. Research has overlooked patient–professional power relations as a factor which may encourage a retrospective re‐evaluation of negative experiences (Edwards *et al*. 2004). Here we consider how the retrospective evaluation of disappointing health care can be understood as part of improving healthcare provision.

Healthcare users who have mental distress (Crow *et al*. 2002, Eriksson and Svedlund 2007), who do not speak the local language fluently (Carmel 1988) or confidently (Rogers *et al*. 2000) are routinely excluded from dis/satisfaction research, with the inclusion of minority languages an exception (Mulcahy and Tritter 1998). The dis/satisfaction of migrants (Almeida *et al*. 2014, Suurmond *et al*. 2011), refugees (Mangrio and Sjögren Forss 2017) and travellers (McGorrian *et al*. 2012) tends to be studied separately from the communities in which they live and access health services (Bradby 2002). The current analysis offers insight into how disappointment plays out in neighbourhoods hosting diverse communities with respect to the full range of services that are being accessed.

## Methods

The article offers an original analysis of primary data from a project which explored the ways in which health care was accessed to develop the concept of healthcare bricolage (Phillimore *et al*. 2018). The project focused on two superdiverse neighbourhoods in each of four European cities Bremen (Germany), Birmingham (UK), Lisbon (Portugal) and Uppsala (Sweden), with the countries selected for their different health, welfare and migration regimes (Sainsbury 2006) (see Table 1). In a parallel sequential methodology we mapped local healthcare facilities and services while talking with locals, then interviewed the local healthcare providers and then residents who were also healthcare users, and finally implemented a household survey. The data on which this article draws were collected between October 2015 and December 2016 from a diverse sample of adults who identified as having had a health concern within the previous 5 years. The sample included healthcare users of diverse migrant status, health status and local language competency, as we made explicit efforts to recruit people who differed from one another, as per maximum diversity sampling. Extended details of neighbourhoods and methods and findings from healthcare provider interviews are published elsewhere (Hamed *et al*. 2018, Phillimore *et al*. 2015, 2019a).

**Table 1:** Characteristics of the comparison countries and neighbourhoods^a^

	City	Health and welfare regimes
**Germany**	**Bremen:** 10^th^ largest city 554646 residents, 30% people from migrant background (deprived and skilled) from 162 countries.	Conservative welfare regime Universal, corporatist healthcare system, decentralised and self‐governing. Compulsory health insurance based on income covers 85% of the population. Direct access to services with choice of provider. Migrants receive a health insurance card allowing access to medical help for acute illness, pain and pregnancy. Without insurance, people must pay or use volunteer doctors, CSOs and welfare organisations. There is no functioning interpretation system. The healthcare ecosystem is very complex so people struggle to understand entitlements. The ecosystem has been transformed into a competitive health market with statutory health insurers behaving as competing corporations. Medical professionals are supposed to report irregular migrants to immigration authorities.
**Portugal**	**Lisbon**: capital and largest city 547733 residents, housing migrants from 172 countries, recent arrival of refugees	Southern European welfare regime Health system is comprised of multiple sectors including a universal national health service (NHS) with co‐payment scheme and exemptions for certain populations. Health subsystems include health insurance for public servants, a growing private insurance health sector and the lottery funded charity‐led parallel health service of Santa Casa da Misericordia (SCML) for vulnerable populations. The economic crisis affected provision and quality of health services as TROIKA imposed severe cuts. Most irregular migrants’ exemptions were removed making access problematic. NHS professionals cannot report irregular migrants to authorities due to professional ethics.
**Sweden**	**Uppsala:** fourth largest city. 202625 residents, people from migrant background from 174 countries (deprived and skilled)	Social Democratic welfare regime Comprehensive universal system. Equity is prioritised through redistributive policies in the form of statutory and municipal taxes, benefits and services aimed at mitigating the damaging effects of poverty. The system of fiscal and non‐fiscal universal benefits, distributed with little means‐testing imply extensive public‐sector employment in health and social care. Health care and welfare available to whole population for a small fee. Only immigrants with legal rights of domicile can access non‐urgent care. Very limited private sector. Provision through for‐profit corporations increasing. Limited austerity since Sweden's major financial crisis and contraction of the welfare state occurred in the 1990s. Emphasis on individual responsibility, healthy living and active lifestyles.
**UK**	**Birmingham**: second largest city. 1073045 residents, 22% foreign born, 47% ethnic minorities from 187 countries.	Liberal welfare regime The UK's NHS introduced as a universal system with primary and secondary healthcare free to all. The past 20 years have seen constant attempts at restructuring to slow down spiraling costs. Shortages of doctors and nurses with the system said to be in crisis and Government refusing to increase the budget. Restructuring in 2013 introduced service commissioning to introduce competition, reduce costs and offer choice for health ‘consumers’. Widespread concerns about capacity to meet rising demand, the exacerbation of recruitment difficulties, reduced investment, long‐term under‐funding of mental health provision and cuts in public health and social care budgets. Immigration legislation denies undocumented migrants and failed asylum seekers free access beyond emergency care. NHS workers are expected to report and refuse to treat undocumented migrants.

Terminology varies by country so data are not comparable. Data for Germany: 2012 national census and Arbeitnehmerkammer: Bericht zur sozialen Lage 2013. Data for Portugal: migrant definition: foreign born and ethnic minorities. Data for Sweden: foreign born and ethnic minorities. Data for the UK: 2011 Census.

This article focuses on findings from interviews with healthcare users. In each country community researchers were recruited for their multi‐lingual abilities and knowledge of local networks, services and non‐government organisations (and subsequently trained using an adapted version of an accredited training model developed by the UK team (Hamed *et al*. 2018, Rodrigues and Padilla 2017)). Community researchers assisted in the recruitment and interviewing of 152 adult residents, around 20 in each neighbourhood, drawing on their social networks, local organisations and snow‐ball sampling from contacts made in the early mapping phase of the research. Interviews were conducted in 30 different languages which included the 4 national languages (English, Portuguese, German and Swedish), well‐established minority languages (Arabic, Somali, Urdu, Bengali, Persian and Hindi), other European languages (Italian, Finnish and French) and relatively newly arrived minority languages (Creole, Tamil, Bosnian, Tigrinya and Nepali). Some residents were interviewed in their second or third language, and a few were accompanied by a relative or friend who offered language support, so the interviews themselves were multi‐lingual. Furthermore, the material is user centred in that the semi‐structured interviews were guided by interviewees’ own concerns and covered all the types of health care used: public, private, digital, informal and transnational.

Maximum variation sampling aimed for heterogeneity in the sample composition in terms of country of origin (including native‐born), age, gender, length of residence, ethnicity, socioeconomic status and language competency. This form of comparison‐focused sampling selects cases based on their difference, to identify factors explaining commonalities and differences (Patton 2002). Any shared traits that emerge do not result from sampling by pre‐determined characteristics and so have greater authenticity and validity.

The project received ethical approval from the relevant committees in each city. After a verbal invitation to participate and the sharing of a participant information form summarising details of the study, interviewees signed consent forms which emphasised the option to stop the interview at any time and to withdraw their data up to 30 days after the interview. Healthcare users were interviewed at the location, and in the language, of their choice and were asked to recall a health concern and describe all actions taken to address it from the emergence of symptoms until some resolution was reached. Interviews lasted between 30 and 120 minutes, averaging about 60 minutes per interview. Prompts such as ‘what did you do next?’ encouraged recall of all actions. All interviews were recorded, transcribed and translated where necessary to English and here all interview excerpts are shown in English.

For the first phase of analysis, data were coded using MAXQDA software and a systematic thematic analytic approach (Guest *et al*. 2011) to identify key issues and actions raised. This involved the transcribed data being repeatedly read by the research team who collectively identified themes. A shared codebook was devised between teams in the four countries using MAXQDA software and the project lead checked inter‐coder reliability across sites.

The expression of dissatisfaction emerged at an early stage in the analysis, with 71, that is, almost half of respondents interviewed, describing disappointing experiences of care. A second round of analysis focused specifically on these experiences. At this stage we extracted all text that had initially been coded under this theme and focused on narratives of disappointing care identifying: (1) what had happened, (2) why it was disappointing, (3) what happened immediately afterwards and (4) what the situation was at the time of the interview as health issues raised by participants were not necessarily resolved (see appendix material). We looked for indications of resources mobilised by healthcare users, which could be material (funds to buy additional care), psychological (confidence; resilience) or social (families, friends, migrant networks). Since dissatisfaction was raised spontaneously in these 71 interviews, there is no implication that the other 89 interviewees were all satisfied, but rather that they did raise dissatisfaction during the interview.

Recruiting interviewees in superdiverse neighbourhoods, without excluding people for being ‘atypical’ or ‘hard to reach or communicate with’, and inviting them to describe the actions taken to address health concerns and the reasons for these actions, meant that the interviews were not structured by professional healthcare providers’ priorities. People had accessed a wide range of types of health care, both in the same locality and from other localities, nationally and internationally. Accounts of disappointing and unsatisfactory health care were offered spontaneously, often in response to being asked why particular actions had been taken to address a specific health concern; perhaps an indication of the importance of such dissatisfaction to the respondents who elected to raise these experiences. The accounts analysed here were explicit verbal descriptions of health care which the interviewee him or herself judged to be poor, inadequate or otherwise unsatisfactory and disappointing.^2^

## Findings

This article reports on the 71 (out of the total 152) respondents who described some aspect of their health care as unsatisfactory or disappointing. The characteristics of respondents expressing dissatisfaction are highly varied (see appendix material). Ages ranged from 18 to over 80 years, with more than 30 countries of birth represented and citizenship from across the regions of Europe, Asia, Africa and America, including 23 born in the case study countries (Germany, Portugal, Sweden, UK). To be clear, this analysis includes both migrants and natives, since the aspects of marginalisation relevant to responding to disappointing care were not confined to migrants. Some 43 were women, 26 said they were Christian, 22 Muslim, with 15 not reporting a faith. Forty respondents reported being in work, 12 were unemployed, 6 were studying and the remaining 13 were retired, unable to work and/or responsible for domestic tasks. In terms of interviewees’ ability to speak the local national language, the full range from native speaker, through fluent and good, to basic and very basic (about 20% of sample) self‐assessed language ability were reported, and while over half of the interviews were undertaken in the local national language, nearly 30 were undertaken in another language.

Since these narratives were spontaneous evaluations made from the service user's perspective, this material was not intended for systematic comparison across healthcare systems. Nonetheless, every neighbourhood included 4 to 12 people describing one or more aspects of their health care as disappointing or unsatisfactory, although negative healthcare experience was not an inclusion criterion for the study. This analysis was conceived as a qualitative investigation, but Table 2 shows the frequencies of socio‐demographic characteristics (gender, migration background and status, employment, local language proficiency and country of residence), of those expressing dissatisfaction and those who did not. The characteristics in Table 2 are almost evenly distributed between the two groups. Thus no strong relationship between single aspects of marginalisation and the expression of disappointment is evident. One possible explanation is that the underlying relationship is complex and involves the intersection of multiple aspects of marginalisation.

**Table 2:** Participant characteristics by dissatisfaction with healthcare services received

Participant characteristics	Total (%) Dissatisfied (n = 71)	Total (%) Not Dissatisfied (n = 81)
Gender		
Male	29 (40.8%)	36 (44.4%)
Female	42 (59.2%)	45 (55.6%)
Migration background		
Born in a different country	47 (66.2%)	50 (61.2%)
Residing in country of birth	24 (33.8%)	31 (38.2%)
Migration status		
Citizen by birth	23 (32.4%)	31 (38.2)
EU National	7 (8.5%)	5 (6.2%)
Naturalised citizen	17 (23.9%)	23 (28.4%)
Permanent status	13 (18.3%)	6 (7.4%)
Refugee status	2 (2.8%)	0 (0%)
Temporary status	4 (5.6%)	6 (7.4%)
Undocumented	0 (0%)	5 (6.2%)
Other/Not Collected	5 (7.0%)	5 (6.2%)
Employment		
Working	40 (56.3%)	31 (38.2%)
Unemployed	13 (18.3%)	19 (23.5%)
Student	6 (8.5%)	3 (3.7%)
Non‐active^a^	12 (16.9%)	28 (34.6%)
Proficiency in language (country of residence)		
Native	37 (52.1%)	39 (48.1%)
Fluent/Good	21 (29.6%)	25 (30.9%)
Basic/Very Basic	10 (14.1%)	13 (16.0%)
None/Not Collected	3 (4.2%)	4 (4.9%)
Country		
Germany	24 (33.8%)	16 (19.8%)
Portugal	15 (21.1%)	27 (33.3%)
Sweden	12 (16.9%)	18 (22.2%)
UK	20 (28.2%)	20 (24.7%)

a Retired, unable to work or domestic tasks.

## Unsatisfactory health care – feeling dismissed

When their attempts to access care were deprioritised, diverted or otherwise ignored by healthcare providers, service users described feeling dismissed and devalued. The vast majority of disappointing encounters reported were with the public health system, with a few cases of dissatisfaction with alternative treatments.

People reported feeling dissatisfied with their health care when symptoms, pain or distress were dismissed(Gro1^3^, Lum34, Mou23, Mou22, Han19, Han 18), the ‘proper tests’ were not undertaken or treatment was not checked in a timely fashion (Edg07, Han11, Gro29, Gro24, Neu7, Neu 19, Sav06, Mou17). Medicines were a particular flashpoint, where feeling dismissed was compounded by a sense that inappropriate medication or dosage might be causing harm (Gro10, Got09, Han 33). Unsatisfactory treatment was usually linked with the failure to resolve health concerns, inadequate communication and a perceived lack of care: as a man in Birmingham said of healthcare professionals: ‘Nobody bothers. It's all talk. For seven years I've been suffering’ (Han17).

A few people attributed feeling dismissed to healthcare providers’ discriminatory attitudes. Several Swedish patients of migrant background felt they might be treated less favourably than native Swedes, although they had little hard evidence (see Bradby *et al*. 2019b) for further discussion). Layla, an Arabic‐speaking woman in Bremen, felt that her daughter was inappropriately dismissed when convulsing with a high fever, and that she received inadequate staff attention because of her appearance and limited language skills:

When they saw me wearing this headscarf, they had the stereotype that I'm ignorant. They don't give me information ‐ I'm like an animal in front of them. Maybe I can't express myself very well in German but I'm not a stupid animal. (Gro09)

Djamila, a Jordanian student in Birmingham, felt that healthcare providers did not take her symptoms seriously because they assumed she was looking for an excuse for failing her exams. Like Layla, she said her feeling of being dismissed was exacerbated by language limitations.

Not only migrants described a sense of unfair discrimination: Orwell, a German native, (Gro42) described being mistaken for an alcoholic when he stumbled into a rural emergency clinic in a great state of confusion. He was eventually diagnosed with a liver abscess, but initially his symptoms were dismissed:

I went to the emergency department […] of course I had not showered for a week, not combed my hair, not shaved, nothing at all somehow and swayed in there so to say. And for them it was immediately clear – ‘Ah yes … an old hippie from the Alb, he has totally drugged himself to death or… drunk himself to death.’ […] And that was a completely wrong diagnosis, yes?

## Components of communication

Patients’ feeling of dismissal was most often linked with poor communication whereby their needs and priorities were said to be ignored. Most of those describing unsatisfactory care spoke the same language as the healthcare providers and did so fluently, but for those who did not speak the local language or had only basic competency, communication problems were exacerbated. Bim, a Sri Lankan man in his 70s who had lived in Germany for 30 years and described his German as ‘good’, said that being misunderstood by doctors was the greatest problem that ‘foreigners’ faced in the public health service:

I'm a patient; he [the doctor] must understand what my problem is. So if they speak generally English then [it is] easy for us to speak to them and easy for them to understand us. With the German, eh, our German he misunderstands, his German we misunderstand, that's why this, especially in the cities, doctors who are treating must know the international language. (Neu22)

This sentiment was echoed by Dimpal, a young Indian woman with temporary residency, who had lived in Lisbon for 4 years and said that ‘whenever we go to the hospital we have a language problem, so I think in Portugal it's the biggest problem’ because ‘all the doctors and nurses know English but they prefer to speak in Portuguese’(Mou12). She felt that the lack of a common language was compounded by a discriminatory attitude against those who could not speak Portuguese, especially if they looked ‘foreign’.

While the need for translation at healthcare encounters has been widely acknowledged, in practice translators are hard to access, not always competent and not necessarily welcomed by health professionals (Got13, Han40). Amani, a young refugee from Sudan who had been in the UK for a year, described how his symptoms remained untreated because his official health service translator failed to lodge the appropriate paperwork and so medication was denied:

The interpreter helped me filling an application to get the medication from the pharmacy. But he was very busy and he has not done the job properly in the end, and we ended up without medication. (Han40)

Having tried to access medication, he then lost hope and gave up trying, despite the persistence of his breathing problems. When communicating in a second or third language and for people consulting with a translator, having a limited time for consultation (Got14) was troublesome. Affran, a 32‐year‐old Kurdish man from Syria, with permanent residency in Sweden, described how healthcare professionals in a hurry disrupted his attempts to recover from the intense anxiety that limited his daily life:

I needed mental health support and more information and that I would communicate with the healthcare professionals better and that they would have had the time to take my problems seriously. I felt that they didn't really care. It was disturbing for me that they didn't have any time. … If I started to talk, talk about something, then they would interrupt me and go to the next point. No time. So I couldn't talk. They were in a hurry. (Sav17)

Affran had limited Swedish (and was fluent in three other languages) but this limitation was only part of the problem, since he felt that staff failed to support him, offering neither enough time, nor a sense of caring about the resolution of his problems.

## Beyond the language barrier

While the absence of a common language was often mentioned, it was far from the only communication problem since healthcare users who were fluent in the local language described similar disappointments. Cristina, a 61‐year‐old woman of Angolan origin, had lived in Lisbon for 39 years, had Portuguese as her mother tongue, and was deeply dissatisfied with her health care. Cristina was diabetic and had both sight problems and leg ulcers that would not heal (Lum 33). She reported the lack of effective treatment for her leg wounds as highly unsatisfactory:

How can I explain? (I would like) that they find what is wrong with my legs. Because there is something bad. … The bad is inside the flesh, the veins, it's there. They should find something to remove what is bad away and then do the treatment. … It is poison that is there and needs to come out. Because it is a wound that heals on the outside but not on the inside.

The interviewer asked ‘You had problems with the doctors?’ to which Cristina replied ‘If there were doctors who could understand our problems, I would already be better.’ Cristina closed the interview by saying:

If they would think a little bit about what I am saying, they would find something. They would be able to take the bad away. There could find what would heal my wound and why it doesn't close. It's not closing because the bad is inside. No one thinks of this.

Cristina, like Affran, felt that her health concerns could be addressed more satisfactorily if only the healthcare professionals would listen more carefully and considerately. The sense of intense dissatisfaction that they both described could not be reduced to a single aspect of the marginalisation that they experienced.

## Responding to dissatisfaction, disappointment and dismissal

When a health concern or symptom was ignored or devalued, people from a wide range of backgrounds felt, not only disappointed at the lack of resolution of the problem, but also personally dismissed (Coyle 1999). Since we asked people to tell the story of their health concern over time, prompting for everything that people did and why, we were able to map how they responded to a negative healthcare experience to understand the meaning of their disappointment.

In what follows we consider responses to disappointing health care and offer two interpretations that are not alternatives, but rather exist in parallel. In many accounts of disappointment and dissatisfaction from our diversity sample, we can see **both** efforts to get access to health care that will effectively address the symptom or condition that prompted the initial consultation **and** strategies to un‐do the sense of dismissal and to avoid it in the future. The extent to which efforts were directed towards accessing care or addressing the existential threat of dismissal varied between dissatisfied healthcare users and over the course of an individual's story.

## Accessing health care and undoing dismissal

Some people responded to their negative experience by focusing on efforts to get more suitable health care. Elsewhere we have described bricolage tactics (Phillimore *et al*. 2019b) (Phillimore *et al*. 2018), whereby healthcare users draw on the resources to which they have access to address their own healthcare needs. The most widespread tactic was to consult a different healthcare provider, either within the public healthcare system or, when people could afford to pay, from a private provider. Consulting another public healthcare professional (Edg15, Mou17, Neu 22, Neu 19) and/or paying for services could mean combining conventional (Edg07, Got14) and alternative treatment (Han23, Han11, Mou03, Mou23, Mou22), sometimes accessed by travelling to another country (Got07, Gro04, Mou03, Lum27). Alternative treatments relieved symptoms that participants said were ignored by the public health system or supported chronic conditions for which healthcare providers only prescribed tablets.

In consulting another professional and/or accessing alternative treatments, people attempted to resolve their health concern. Goldi, a Turkish‐born German woman in her thirties who had an aneurysm, explained that when rehabilitative health services are paid for privately, ‘the doctors treat you really differently: they're much more open; they really support their patients; they take the trouble’ (Gro04). Getting access to appropriate services was clearly an end in itself, particularly with life‐threatening conditions. However, the satisfaction of being proven correct in having one's symptoms acknowledged and taken seriously was also important in how people responded. This was clear in the story of Zoya in Bremen whose severe abdominal pain was dismissed by the walk‐in emergency clinic. Having been sent home, she consulted the internet about the tests she had been given in the hospital and returned to the clinic the next morning to re‐present her symptoms, whereupon she had an appendectomy (Neu11).

Persuading a professional who had dismissed one's symptoms to acknowledge that they were, in fact, significant was important for Djamila, in Birmingham, who insisted on a referral to a specialist eye clinic:

I insisted that she have a second look … ‘if you don't see my eye’ – my Arab roots came up – so I told her ‘if you don't see my eye I'll go to the reception and I'll tell them … that I'm not very satisfied with the service.’ So she's like ‘Alright … just to let you know there is nothing wrong I am going to get the nurse to have another look at your eye.’ And when the nurse came in, she was like ‘Why don't you put the dye?’ Something with colour – iodine I think – like yellowish. As soon as she put the drop in my eye the little air bubble emerged and she was like ‘There it is!’ I was like ‘See!’

Djamila's triumphant ‘See!’ when her eye problem was confirmed, sums up the sense of being personally vindicated. The validation of overturning an initial dismissal was described in the two formal complaints that were described to us (Mou45, Neu21). More common though, in our interviews, was to give up – either totally or partially – on engaging with the public healthcare system.

Unsatisfactory health care meant that some people said that in the future they would avoid healthcare providers at all costs (Han11, Sav11, Got12). In refusing the public health system, some took up alternative treatments, supplements and/or therapies to maintain their health or alleviate symptoms: ‘you've got to become your own doctor’, we were told by Uwe in Bremen (Neu06).

The various responses to dissatisfactory health care – seeking out alternatives, seeking vindication, rejecting the public healthcare system – could of course overlap within a single case. Having experienced disappointing care, a person might resolve their health concern by consulting alternative practitioners **and** by persisting with the public healthcare system, before eventually having another negative encounter, leading to withdrawal. The multiple and overlapping responses to disappointing care are illustrated by Carol, a British Black Caribbean woman in her mid 40s, who had suffered a whiplash injury 20 years earlier. She described the different ways that she had coped with ongoing back and neck problems, including multiple consultations with both NHS and private practitioners. Carol had both negative and positive experiences of public health care and of the chiropractors that she had hired privately. She had recently been offered a surgical intervention for chronic pain but had turned it down, since she felt it was too risky. She described how she had learned to cope with her painful symptoms:

I became an aromatherapist and an Indian head masseur. I learned how to do those therapies and I wanted to work for myself. I was in so much pain I wanted to find an alternative to painkillers and medication. [And] I have got to smoke my weed. The two go hand in hand ‐ I believe it is pain relief. I feel relaxed. I think if I didn't have it I would be grumpy because I would be uncomfortable. (Han23)

When asked which of her healthcare professionals she trusted most, Carol replied ‘I don't trust none of them’, because ‘everybody has got a caseload that is perhaps too heavy’ and, consequently ‘you have to push for everything, everything ‐ everybody needs a little push!’ Carol refused the idea that she faced barriers to accessing health care, saying ‘there are no barriers because I am finding ways around!’ She described strategies for accessing alternative and cut‐price therapies and attributed her ability to both push the healthcare professionals and find alternatives to her previous employment experience as a support worker for homeless people. Despite saying that she could overcome the difficulties of the over‐stretched healthcare system by pushing for alternatives, at the end of the interview Carol acknowledged that despite repeated attempts to persuade her doctor to refer her for occupational therapy, ‘I haven't heard from him for months.’

Carol, like Cristina in Lisbon, was unemployed with a chronic health problem that was unresolved despite repeated interventions over decades. But Cristina's response to her disappointing care differed from Carol's: when asked about alternative treatments she rejected the idea of acupuncture, saying ‘nowadays I am scared of everything’. Although Cristina said ‘I would love to have someone who could support me in the future to understand how I could get well again’, she said there were ‘a lot of crazy people around but there is no one who helps’. Both Carol and Cristina described their dissatisfaction and disappointment with health care; and while Carol continued to engage with service provision to address her problems believing in her own effectiveness, Cristina was disillusioned with her healthcare providers and withdrawn.

## Healthcare context

Carol in Birmingham and Cristina in Lisbon were both seeking health care in systems that had been effected by austerity‐driven cuts over the years prior to our interviews with them. In these and other interviews, healthcare users described the economic (Edg07, Neu31) and political context (Mou22) as reasons for disappointing health care: as Carol noted ‘everybody has got a caseload that is perhaps too heavy’. Systemic problems, when noted, were offered as a reason for why healthcare professionals had failed to meet individual needs. Under neo‐liberal regimes that drive the cost‐cutting of public services across Europe (Legido‐Quigley *et al*. 2016, Napier *et al*. 2014), responsibility for accessing appropriate services is moved from collective and professional bodies to the individual's initiative, without regard for how well equipped that person is to take on such work. This tension between individual and collective responsibility for health care is part of what is at stake in considering how patients respond to disappointing health care. While Carol told us she could be strategic in her dealings with overburdened healthcare professionals, Cristina felt that she had been failed and that she had absolutely no recourse to address this failure as an unemployed, older widow.

In the following discussion we contextualise the expressions of, and response to, disappointing health care in the wider analyses of this research project and more generally in campaigns for patient participation.

## Discussion

People's emotional and practical responses to their symptoms and the responsiveness of healthcare providers to their needs matter, both for individual healthcare users’ wellbeing and for increasing care quality (Coulter *et al*. 2014). Research into dissatisfaction with health care tends to sample particular clinical populations, excluding those marginalised by limited language, uncertain migration status, mental illness and poverty (Hill 2010). While the incorporation of healthcare users’ experience (Barry *et al*. 2001) is accepted in principle, the systematic and structured incorporation of patients’ views remains elusive even in those European countries with active user movements (Rozenblum *et al*. 2013). The introduction of standards informed by users’ negative experiences is a well‐established mechanism for improving health care, but is nonetheless a normative strategy for improving access to, and quality of, health services: normative standards may never entirely meet the needs of societies that are not only diverse, but also diversifying (Phillimore *et al*. 2019c).

Our multi‐national study, with maximum diversity samples from eight neighbourhoods, collected accounts of negative healthcare experiences from a range of users, some of whom are regularly under‐represented in survey research, highlighting aspects of service provision where people want greater responsiveness. Our findings echo existing research in that having one's symptoms or priorities ignored, being offered inadequate time, respect or care were experienced, not only as disappointing, but also as existentially threatening. The process through which people assess their experience as unsatisfactory and attribute responsibility for their disappointment to individuals and organisations could not be attributed solely to demographics or particular, identifiable barriers. As with existing research on negative health encounters (Coyle 1999, Werner and Malterud 2003) the individual complexity of accounts of disappointing health care precludes simple explanation.

Responses to dissatisfactory health care included seeking out alternative provision within and beyond the public system, both for more suitable treatment and for vindication of the initial dismissal. For some people, the experience of disappointing health care involved aspects of marginalisation, including the inability to pay for private services, and/or the inability to speak the local language and/or to navigate the healthcare system (Green *et al*. 2014). But we also heard from disappointed healthcare users who spoke the local language fluently and could afford to pay for additional services.

With the reduction in the reach of the public healthcare system, individuals carry the responsibility, often financial, of getting access to services in a timely fashion as illustrated by Carol in Birmingham who emphasised her own ability to strategise. Cristina in Lisbon felt unable to insist on services to meet her needs and, lacking the means to pay for private care, described a sense of hopeless abandonment. Uwe in Bremen felt that the only appropriate response was to become his own doctor.

While low wages or limited language could impede attempts to address disappointing health care, our qualitative analysis of data in Table 2 suggests no clear pattern of how aspects of marginalisation play out in different countries. Our quantitative analyses of a household survey found that the welfare and healthcare context and individual level characteristics including gender, level of education and migration background affect the bricolage tactics that healthcare users adopted (Phillimore *et al*. 2019b). However, such patterns were not apparent in analysing the spontaneous expressions of disappointment in the interview sample. Our research design and diversity sampling by neighbourhood allowed us to identify aspects of marginalisation that were relevant to particular cases, but did not support an analysis of causal relations. We expected features of the neighbourhoods to be more apparent in the interviews than turned out to be the case: healthcare users’ efforts to engage appropriate health care were not confined to the locality, and extended beyond its boundaries in finding services digitally, nationally and transnationally. The configuration of locally available services did not feature much in healthcare users’ accounts of their negative experiences.

Our analysis suggests that any intervention to improve both access to and the quality of care and to reduce the expression of dissatisfaction, needs to address the responsiveness of healthcare provision rather than to target groups of healthcare users as defined by a particular characteristic thought to indicate marginalisation. A more radically responsive and pro‐actively equitable service provision that considers healthcare users’ differential ability to advocate and organise for themselves, remains an ideal, although not one that had been arrived at in the eight neighbourhoods that we studied.

Our interviews were carried out at the end of 2015 and throughout 2016, when significant numbers of refugees were arriving into Southern Europe via the Mediterranean and Balkan routes and the politics of welfarism and nativism were shaping national policies on welfare access. Ultimately, the imperative to create equity standards that attend to the experiences and priorities of populations characterised by globalised migration, and ‘the necessity of changing existing practices that are potentially discriminatory and exclusive’ are political decisions (Cattacin *et al*. 2013: 256). The link between wider democratic structures and healthcare systems’ ability to respond to the values and priorities of those using the services, may underlie the lack of meaningful change in incorporating users’ view systemically (Beresford 2019).

Understanding how to design services that are sufficiently robust to maintain standards and respond to the range of needs and priorities that a diversified and diversifying population presents is an urgent task if national healthcare systems are to maintain the ideal of universal provision on the basis of need. Offering health care that is responsive to healthcare users and their priorities, including their search for alternative services, implies healthcare providers’ increased responsiveness that works against the normativity of asserting quality standards. The balancing out of normative quality standards at the national and European levels against responsiveness to healthcare users’ own assessment of their needs is a cultural and a political question which requires our attention.

## Supporting information


**Table S1**: Select characteristics of interviewees who expressed dissatisfaction with healthcare services

## Notes

1 Our study focuses on ‘service users’ to encompass the range of formal and informal health care and other services taken up by a single person, but the narrower term ‘patients’ is used in the research under discussion.

2 This contrasts with another analysis where healthcare users explicitly expressed satisfaction with their health care, although we assessed the services that they described as being inadequate (Bradby *et al*. 2018).

3 These codes are included for the authors’ ability to trace the evidence through a big data set, and correspond to the ID in the appendix.
