# Supplementary material for: ‘If there were doctors who could understand our problems, I would already be better’: dissatisfactory health care and marginalisation in superdiverse neighbourhoods
Source: Sociol Health Illn. 2020 Feb 4;42(4):739–57. doi: 10.1111/1467-9566.13061 (PMC7318273; doi:10.1111/1467-9566.13061)
Supplement: Supplementary file 1 — Table S1: Select characteristics of interviewees who expressed dissatisfaction with healthcare services [file SHIL-42-739-s001.docx]

Supplementary material: select characteristics of interviewees who expressed dissatisfaction with healthcare services

| **ID** | **Age** | **Gender** | **Marital Status** | **Country of birth** | **Region of citizenship** | **Faith** | **Employment** | **National Language**  **Ability** | **Language**  **during interview** | **Second language** |
| --- | --- | --- | --- | --- | --- | --- | --- | --- | --- | --- |
| Gro01 | 30-44 | F | Married | Germany | Western Europe | Muslim | Working | Fluent | German | Turkish |
| Gro03 | 30-44 | F | Married | Cameroon | Middle Africa | Christian | Working | Fluent | German | English |
| Gro04 | 30-44 | F | Single | Turkey | Western Asia | Muslim | Unable to work | Fluent | German | Turkish |
| Gro07 | 45-59 | F | Single | Poland | Western Europe | Christian | Working | Fluent | German | Polish |
| Gro09 | 45-59 | F | Married | Tunisia | Northern Africa | Muslim | Domestic tasks | Good | Arabic | None |
| Gro10 | 30-44 | F | Married | Bulgaria | Eastern Europe | Muslim | Working | Basic | Turkish | Bulgarian |
| Gro13 | 18-29 | M | Single | Kurdistan | Western Asia | Muslim | Unemployed | Good | German | Arabic |
| Gro24 | 30-44 | M | Married | Bulgaria | Eastern Europe | Christian | Working | None | Bulgarian | Romani |
| Gro25 | 30-44 | M | Divorced | Not recorded | Western Africa | Muslim | Unemployed | Fluent | German | French |
| Gro29 | 30-44 | F | Married | Cameroon | Middle Africa | Christian | Working | Fluent | German | French |
| Gro33 | 30-44 | F | Married | Germany | Western Europe | Christian | Working | Native | German | None |
| Gro39 | 45-59 | M | Not recorded | Germany | Western Europe | None | Unemployed | Native | German | None |
| Gro42 | 45-59 | M | Married | Germany | Western Europe | None | Working | Native | German | English |
| Neu06 | 30-44 | M | Single | Ghana | Western Africa | Muslim | Working | Native | German | English |
| Neu11 | 30-44 | F | Married | Russia | Eastern Europe | Christian | Working | Fluent | German | Russian |
| Neu15 | 30-44 | M | Single | Mexico | Central America | None | Unemployed | Basic | Spanish | German |
| Neu17 | 18-29 | F | Married | Germany | Western Europe | Christian | Student | Native | German | English |
| Neu18 | 45-59 | F | Divorced | Germany | Western Europe | None | Working | Native | German | English |
| Neu19 | 45-59 | M | Divorced | Germany | Western Europe | Christian | Retired | Native | German | None |
| Neu22 | 60-79 | M | Married | Sri Lanka | Southern Asia | Hindu | Retired | Good | Tamil | English |
| Neu31 | 45-59 | M | Single | Turkey [?] | Western Europe | None | Working | Fluent | German | Turkish |
| Neu32 | 30-44 | F | Married | Russia | Eastern Europe | Christian | Unemployed | Fluent | German | Russian |
| Neu41 | 45-59 | F | Married | Germany | Western Europe | Christian | Working | Native | German | English |
| Lum27 | 30-44 | F | Single | Sao Tome | Southern Europe | Not recorded | Working | Native | Portuguese | none |
| Lum30 | 30-44 | F | Single | Portugal | Southern Europe | None | Unemployed | Native | Portuguese | English |
| Lum32 | 30-44 | M | Married | India | Southern Asia | Hindu | Working | Basic | Hindi | Gujarati |
| **ID** | **Age** | **Gender** | **Marital Status** | **Country of birth** | **Region of citizenship** | **Faith** | **Employment** | **National Language**  **Ability** | **Language during**  **interview** |  |
| Lum33 | 60-79 | F | Widowed | Angola | Southern Europe | Christian | Working | Native | Portuguese | None |
| Lum34 | 30-44 | M | Single | Angola | Southern Europe | Christian | Working | Native | Portuguese | English |
| Lum35 | 30-44 | F | Single | Portugal | Southern Europe | Christian | Working | Native | Portuguese | English |
| Lum36 | 30-44 | F | Married | Portugal | Southern Europe | Christian | Working | Native | Portuguese | None |
| Lum41 | 18-29 | M | Single | Portugal | Southern Europe | Christian | Working | Native | Portuguese | Spanish |
| Mou04 | 45-59 | F | Married | Portugal | Southern Europe | Christian | Unemployed | Native | Portuguese | None |
| Mou08 | 18-29 | M | Single | Bangladesh | Southern Asia | Not recorded | Working | Good | Bengali | Hindi |
| Mou10 | 80+ | F | Widowed | Portugal | Southern Europe | Christian | Retired | Native | Portuguese | None |
| Mou12 | 30-44 | F | Married | India | Southern Asia | Ravidassian | Domestic tasks | Very Basic | Punjabi | Hindi |
| Mou14 | 18-29 | M | Single | Portugal | Southern Europe | Not recorded | Working | Native | Portuguese | None |
| Mou17 | 30-44 | F | Divorced | Brazil | South America | Christian | Working | Native | Portuguese | Spanish |
| Mou23 | 45-59 | F | Divorced | China | Eastern Asia | Not recorded | Working | Native | Chinese | Portuguese |
| Got03 | 18-29 | M | Single | Syria | Western Asia | Muslim | Unemployed | Basic | Arabic | Swedish |
| Got07 | 18-29 | M | Single | Eritrea | Eastern Africa | Christian | Student | Basic | Tigrinya | Amharic |
| Got09 | 30-44 | M | Married | Palestine | Northern Europe | Muslim | Working | Fluent | Arabic | Swedish |
| Got10 | 60-79 | F | Divorced | Sweden | Northern Europe | Christian | Working | Native | Swedish | English |
| Got12 | 45-59 | F | Married | Kenya | Northern Europe | Christian | Working | Fluent | English | Swedish |
| Got13 | 60-79 | F | Widowed | Iran/Persia | Northern Europe | Muslim | Domestic tasks | Basic | Farsi | Swedish |
| Got14 | 45-59 | F | Married | (Kurdish) | Northern Europe | Muslim | Working | Fluent | Swedish | Kurdish |
| Got16 | 80+ | M | Married | Indonesia | Northern Europe | None | Retired | Fluent | English | Swedish |
| Sav01 | 30-44 | F | In a Relationship | Sweden | Northern Europe | Christian | Working | Native | Swedish | None |
| Sav06 | 60-79 | F | Divorced | Somalia | Eastern Africa | Muslim | Working | Fluent | Somali | Swedish |
| Sav12 | 30-44 | F | Married | Sweden | Northern Europe | None | Unemployed | Native | Swedish | English |
| Sav17 | 45-59 | M | Single | Syria | Western Asia | Muslim | Student | Basic | Arabic | Not collected |
| Edg06 | 30-44 | F | Single | UK | Northern Europe | None | Working | Fluent | Cantonese | None |
| Edg07 | 18-29 | M | Single | Lithuania | Eastern Europe | None | Working | Fluent | Russian | Polish |
| **ID** | **Age** | **Gender** | **Marital status** | **Country of birth** | **Region of citizenship** | **Faith** | **Employment** | **National language ability** | **Language during**  **interview** |  |
| Edg15 | 18-29 | F | Single | UK | Northern Europe | None | Student | Native | English | Not Collected |
| Edg26 | 18-29 | F | Single | Poland | Northern Europe | None | Working | Fluent | Swedish | Polish |
| Edg27 | 18-29 | F | Engaged | Jordan | Western Asia | Islam | Student | Fluent | Arabic | French |
| Edg31 | 60-79 | F | Divorced | USA | Northern Europe | Christian | Working | Native | English | None |
| Edg32 | 30-44 | F | Married | France | Western Europe | None | Working | Fluent | French | Spanish |
| Edg35 | 45-59 | M | Married | Hong Kong | Northern Europe | None | Working | Basic | Cantonese | None |
| Han10 | 30-44 | F | Single | Vietnam | Northern Europe | None | Domestic tasks | Fluent | Mandarin | None |
| Han11 | 18-29 | M | Married | Pakistan | Northern Europe | Islam | Working | Fluent | Urdu | Punjabi |
| Han12 | 60-79 | M | Separated | Pakistan | Northern Europe | Islam | Retired | Fluent | Urdu | Punjabi |
| Han13 | 30-44 | F | Divorced | UK | Northern Europe | Islam | Student | Fluent | Not recorded | Not Collected |
| Han17 | 60-79 | M | Separated | Pakistan | Western Europe | Islam | Domestic tasks | Basic | Urdu | Dutch |
| Han18 | 18-29 | M | Married | UK | Northern Europe | Islam | Working | Fluent | Bengali | None |
| Han19 | 45-59 | F | Divorced | Kenya | Northern Europe | Islam | Unemployed | Fluent | Not recorded | Not Collected |
| Han23 | 45-59 | F | Single | UK | Northern Europe | Christian | Unemployed | Native | None | None |
| Han24 | 30-44 | F | Separated | Rwanda | Northern Europe | Christian | Working | Fluent | French | Swahili |
| Han28 | 30-44 | M | Single | UK | Northern Europe | Christian | Working | Native | English | None |
| Han33 | 30-44 | M | Married | Ghana | Northern Europe | Muslim | Working | Fluent | French | None |
| Han40 | 18-29 | M | Single | Sudan | Northern Africa | Muslim | Unemployed | Basic | Arabic | None |
